# TNF-α sensitizes chemotherapy and radiotherapy against breast cancer cells

**DOI:** 10.1186/s12935-017-0382-1

**Published:** 2017-01-23

**Authors:** Xiao Wu, Meng-Yao Wu, Min Jiang, Qiaoming Zhi, Xiaojie Bian, Meng-Dan Xu, Fei-Ran Gong, Juan Hou, Min Tao, Liu-Mei Shou, Weiming Duan, Kai Chen, Meng Shen, Wei Li

**Affiliations:** 1 Department of Oncology, The First Affiliated Hospital of Soochow University, Suzhou, 215006 China; 2 Department of General Surgery, The First Affiliated Hospital of Soochow University, Suzhou, 215006 China; 3 Department of Hematology, The First Affiliated Hospital of Soochow University, Suzhou, 215006 China; 4 Department of Oncology, the People’s Hospital of Jingjiang, Jingjiang, 214500 China; 5 PREMED Key Laboratory for Precision Medicine, Soochow University, Suzhou, 215021 China; 6 Jiangsu Institute of Clinical Immunology, Suzhou, 215006 China; 7 Institute of Medical Biotechnology, Soochow University, Suzhou, 215021 China; 8 Center for Systems Biology, Soochow University, Suzhou, 215006 China; 9 Department of Oncology, The First Affiliated Hospital of Zhejiang Chinese Medicine University, Hangzhou, 310006 China

**Keywords:** TNF-α, Chemotherapy sensitization, Radiotherapy sensitization, Breast cancer

## Abstract

**Purpose:**

Despite new developments in cancer therapy, chemotherapy and radiotherapy remain the cornerstone of breast cancer treatment. Therefore, finding ways to reduce the toxicity and increase sensitivity is particularly important. Tumor necrosis factor alpha (TNF-α) exerts multiple functions in cell proliferation, differentiation and apoptosis. In the present study, we investigated whether TNF-α could enhance the effect of chemotherapy and radiotherapy against breast cancer cells.

**Methods:**

Cell growth was determined by MTT assay in vitro, and by using nude mouse tumor xenograft model in vivo. Cell cycle and apoptosis/necrosis were evaluated by flow cytometry. DNA damage was visualized by phospho-Histone H2A.X staining. mRNA expression was assessed by using real-time PCR. Protein expression was tested by Western blot assay.

**Results:**

TNF-α strengthened the cytotoxicity of docetaxel, 5-FU and cisplatin against breast cancer cells both in vitro and in vivo. TNF-α activated NF-κB pathway and dependently up-regulated expressions of CyclinD1, CyclinD2, CyclinE, CDK2, CDK4 and CDK6, the key regulators participating in G1→S phase transition. As a result, TNF-α drove cells out of quiescent G0/G1 phase, entering vulnerable proliferating phases. Treatment of TNF-α brought more DNA damage after Cs^137^-irradiation and strengthened G2/M and S phase cell cycle arrest induced by docetaxel and cisplatin respectively. Moreover, the up-regulation of RIP3 (a necroptosis marker) by 5-FU, and the activation of RIP3 by TNF-α, synergistically triggered necroptosis (programmed necrosis). Knockdown of RIP3 attenuated the synergetic effect of TNF-α and 5-FU.

**Conclusion:**

TNF-α presented radiotherapy- and chemotherapy-sensitizing effects against breast cancer cells.

## Background

Breast cancer is by far the most frequent cancer among women worldwide and accounts for 30% of all new cancer cases in females with a greater incidence in women over the age of 60 years [1–5]. Although a variety of treatments including surgical resection, adjuvant chemotherapy, hormonal therapy, and radiotherapy have been shown to improve survival and reduce the risk of tumor reoccurrence, breast cancer remains as one of the leading causes of death in women [1–5]. Chemotherapy, as a conventional method of cancer treatment, is generally performed by using cytotoxic agents. Radiotherapy plays a vital role in local treatment of lymph node metastasis in breast cancer. However, chemotherapy and radiotherapy have effects on both normal and tumor cells, which means patient have to suffer side effects that come along, and the toxicity is always dose dependent [1, 5]. Therefore, finding ways to reduce the dose of chemotherapy and radiotherapy, without affecting their therapeutic efficiency, is particularly important to tumor therapy.

Tumor necrosis factor alpha (TNF-α) is a classical member of a ligand family of cytokines that include TNF, lymphotoxin-α (LTα), Fas ligand (FasL), CD40 ligand (CD40L), and TNF-related apoptosis-inducing ligand (TRAIL) [6]. TNF-α is a pleiotropic pro-inflammatory cytokine inducing a broad range of cellular responses, ranging from inflammatory cytokine production, cell survival, cell proliferation, cell differentiation and cell death. TNF-α can trigger different forms of PCD (programmed cell death) that are morphologically distinguished as apoptosis and necroptosis [7]. Lots of studies have confirmed that TNF-α can recruit a variety of signaling molecules to induce apoptosis and necroptosis to exert its cytotoxicity through binding to TNF-α receptors, including TNF receptor-associated death domain (TRADD), TNF receptor-associated factor 2 (TRAF2), and receptor-interacting protein kinase 1 and 3 (RIP1 and RIP 3) [8]. Therefore, TNF-α has been used as an antitumor agent previously because of its broad spectrums of cytotoxic effects against a variety of cancer cells, including colorectal cancer, melanoma and sarcoma [9–11]. However, the clinical application of TNF-α has been limited, largely due to its induction of pro-inflammatory and anti-apoptotic gene transcription mainly via activating the NF-κB signal pathway, which leads to systemic toxicity. Recently, studies have shown that combination with low-dose TNF-α could enhance therapeutic effects of chemotherapeutic drugs [12, 13].

In our present study, we investigate whether TNF-α could enhance the cytotoxicity of chemotherapeutics and radiotherapy against breast cancer cells. The mechanisms involved in the synergistic anticancer effect were also investigated.

## Methods

### Cell line and cultures

Human breast cancer cell lines MDA-MB-231 and MCF-7 were purchased from American Type Culture Collection (ATCC, VA, USA). Cells were maintained in RPMI-1640 medium (Gibco, NY, USA), supplemented with 10% fetal calf serum (Gibco), 100 units/ml penicillin, and 100 mg/ml streptomycin. The cultures were incubated at 37 °C in a humidified atmosphere with 5% CO_2_. Cells were passaged every 2–3 days to obtain exponential growth.

### Reagents

TNF-α was purchased from Sigma (St Louis, Missouri, USA). Docetaxel was purchased from Jiang Su Heng Rui Medicine CO., LTD. (Liangyungang, Jiangsu, China). 5-Flurouracil (5-FU) was purchased from Shanghai Xudong Haipu Pharmaceutical CO., LTD. (Shanghai, China). Cisplatin was purchased from Nanjing Pharmaceutical Factory CO., LTD. (Nanjing, Jiangsu, China). Bay11-7082 was purchased from Sigma (St. Louis, MO, USA).

### MTT assay

Cellular growth was evaluated by MTT (methyl thiazolyl tetrazolium) assay [14]. Cells were seeded into 24-well tissue culture plates at 5 × 10^4^ cells per well. After treatment, MTT (Sigma) was added to each well to a final concentration of 0.5 mg/ml, followed by incubation at 37 °C for 4 h. The medium was then removed, and 800 μl of dimethyl sulfoxide (DMSO) was added per well. The absorbance in each well was measured at 490 nm using a microplate ELISA reader (Bio-Rad Laboratories, CA, USA). The relative cell viability was calculated as follows: relative cell viability = (mean experimental absorbance/mean control absorbance) ×100%. The concentration that caused 50% growth inhibition (IC_50_) was calculated by the modified Kärbers method [14] according to the formula: IC_50_ = lg^−1^ [Xk − i (∑p − 0.5)], in which Xk represents the logarithm of the highest drug concentration; i is that of ratio of adjacent concentration; and ΣP is the sum of the percentage of growth inhibition at various concentrations.

### Nude mouse tumor xenograft model and treatment

Four-week-old female BALB/c athymic nude mice were purchased from Shanghai SLAC Laboratory Animal Co. Ltd (Shanghai, China) and received humane care according to the Soochow University Institutional Animal Care and Treatment Committee. MDA-MB-231 cells were injected into the left flanks of the mice in a total volume of 100 μl (0.5 × 10^7^ cells). TNF-α (20 ng each injection, intratumorally) [15], cisplatin (2 mg/kg per body weight, intraperitoneally) [16], and docetaxel (2 mg/kg per body weight, intravenously) [17] were administered every three days. 5-FU was administered intraperitoneally (10 mg/kg per body weight) everyday. Treatments were conducted for 20 days.

### Cell cycle analysis

Cell cycle analysis using propidium iodide (PI, Sigma) was performed as previously described [14]. Prior to treatment, cells were synchronized in the cell cycle by serum starvation for 24 h. After treatment, the cells were fixed in 80% ice cold ethanol, and incubated with 0.5% Triton X-100 solution containing 1 mg/ml RNase A at 37 °C for 30 min. PI was added to a final concentration of 50 μg/ml followed by 30 min incubation in the dark. Cellular DNA content was analyzed by a fluorescence-activated cell sorter (FACS; Becton–Dickinson, NJ, USA). Data were processed by ModFit LT software (Verity Software House, ME, USA).

### Real-time PCR

Total RNA was extracted using Trizol reagent (Invitrogen, CA, USA) according to the manufacturer’s protocol. After spectrophotometric quantification, 1 μg total RNA in a final volume of 20 μl was used for reverse transcription with PrimeScript RT Reagent Kit (TaKaRa, Otsu, Shiga, Japan) according to the manufacturer’s protocol. Aliquots of cDNA corresponding to equal amounts of RNA were used for quantification of mRNA by real-time PCR using the LightCycler 96 Real-time Quantitative PCR Detection System (Roche, Indianapolis, IN, USA). The reaction system (25 μl) contained the corresponding cDNA, forward and reverse primers, and SYBR Green PCR master mix (Roche). All data were analyzed using β-actin gene expression as an internal standard. The specific primers were as follows: (1) Cyclin D1, forward, 5′-GCATCTACACCGACAACTCCAT-3′, reverse, 5′-GTTTGTTCTCCTCCGCCTCT-3′, product, 153 bp; (2) Cyclin D2, forward, 5′-CTGAATACTCCTCCCCTCTTCTCTT-3′, reverse, 5′-TCAGCAGGAAGGGGTGTCTCT-3′, product, 142 bp; (3) Cyclin D3, forward, 5′-CAGCCAGACCAGCACTCCTAC-3′, reverse, 5′-GAAGGGGGAACAGACGCC-3′, product, 162 bp; (4) Cyclin E, forward, 5′-AAGGTTTCAGGGTATCAGTGGTG-3′, reverse, 5′-TGCTCGGGCTTTGTCCAG-3′, product, 183 bp; (5) CDK2, forward, 5′-AATAGGCTGGGAGACTGAAGACT-3′, reverse, 5′-ATCCTTGGCAACTGAACAACTAA-3′, product, 228 bp; (6) CDK4, forward, 5′-TGGAGTGTTGGCTGTATCTTTGCT-3′, reverse, 5′-GCAGCCCAATCAGGTCAAAG-3′, product, 106 bp; (7) CDK6, forward, 5′-CCCTGTCTCACCCATACTTCC-3′, reverse, 5′-GCCTCCAGATAGCAATCCTCC-3′, product, 204 bp; (8) β-actin, forward, 5′-TCATGAAGTGTGACGTGGACAT-3′, reverse, 5′-CTCAGGAGGAGCAATGATCTTG-3′, product, 158 bp.

### Immunofluorescence

Cells were 4% formaldehyde fixed (10 min) at 0.5 h post 40 Gy Cs^137^-irradiation and then incubated with 1% BSA and 0.3% triton for 1 h to permeabilise the cells and block non-specific protein–protein interactions. The cells were then incubated with the Phospho-Histone H2A.X (Ser139) (20E3) Rabbit antibody (Cell Signaling Technology, Beverly, MA, USA) (1:400) overnight at 4 °C. The secendary antibody was Alexa Fluor^®^ 488 Conjugated anti-rabbit (Cell Signaling Technology, Beverly, MA, USA) IgG (H+L) used at a 1:2000 dilution for 1 h. DAPI was used to stain the cell nuclei (blue). Fluorescence signals were taken with Leica fluorescence microscope DM4000 (Leica Wetzlar GmbH, Germany).

### Luciferase reporter gene assay

The reporter plasmid, pNF-κB-luc, containing the κB-enhancer consensus sequences [(TGGGGACTTTCCGC) ×5] and NF-κB-dependent firefly luciferase gene [18] was purchased from Stratagene (La Jolla, CA, USA). The internal control plasmid, pRL-SV40, which contains the renilla luciferase gene, was obtained from Promega (Madison, WI, USA). Cells were transiently cotransfected with the reporter plasmid (500 ng/well) and the pRL-SV40 plasmid (100 ng/well) for 8 h using Lipofectamine 3000 according to the protocol of the manufacturer. The medium was then renewed and treatments were started. After treatment, the cell lysates were subjected to the dual luciferase reporter assay (Promega) according to the recommendations of the manufacturer and luciferase activities were measured with the GloMax-20/20 luminometer (Promega). The results were expressed as relative luciferase activity, which is the ratio of firefly luciferase activity to renilla luciferase activity.

### Apoptosis assays

Apoptosis was evaluated using the Annexin V-FITC/PI Apoptosis Detection kit (Biouniquer Technology, Nanjing, China) according to the manufacturer’s instructions [14]. The cells were resuspended in binding buffer, and Annexin V-FITC and propidium iodide (PI) were added to the buffer and incubated at room temperature for 15 min in the dark, followed by flow cytometry using a Beckman Coulter FC500 dual-laser five-color flow cytometer (Beckman Coulter, Fullerton, CA, USA).

### Western blot analysis

Total protein was extracted using a lysis buffer containing 50 mM Tris–HCl (pH 7.4), 150 mM NaCl, 1% Triton X-100, 0.1% sodium dodecyl sulfate (SDS), 1 mM EDTA, protease inhibitors (10 mg/ml leupeptin, 10 mg/ml aprotinin, 10 mg/ml pepstatin A, 1 mM 4-[2-aminoethyl] benzenesulfonyl fluoride), and phosphatase inhibitors (1 mM NaF, 1 mM Na3VO4). The protein extracts were separated by 10% SDS–polyacrylamide gel electrophoresis (PAGE) and were transferred to polyvinylidene fluoride (PVDF) membranes (Millipore, St Charles, MO, USA). After 1-h blocking in 5% non-fat milk, the membranes were incubated overnight with primary antibodies, rabbit anti-RIP3 (Cell Signaling Technology) or mouse anti-β-actin (Santa Cruz Biotechnologies, CA, USA), at 4 °C. Protein expression was determined using horseradish peroxidase—conjugated antibodies followed by enhanced chemiluminescence) (ECL) detection (Millipore). Protein band analysis was performed using Quantity One 4.6.2 software (Bio-Rad Laboratories, CA, USA). β-actin was used as internal control.

### Transfection of small interfering RNA

Target specific small interfering RNAs (siRNAs) were synthesized by Genepharma (Shanghai, China). The specific sequences were as follows: (1) Control-siRNA, 5′-UUCUCCGAACGUGUCACGUdTdT-3′; (2) RIP3-siRNA-1, 5′-UAACUUGACGCACGACAUCAGGCUGUU-3′; (3) RIP3-siRNA-2, 5′-GCAGUUGUAUAUGUUAACGAGCGGUCG-3′ [19]. The transfections were performed with siRNA-Mate Transfection Reagent (Genepharma) according to the manufacturer’s protocol.

### Statistical analysis

Each experiment was performed a minimum of three times. Results were expressed as the mean value ± standard deviation (SD). Statistical analysis was performed by unpaired Student’s *t* test. A *P* value <0.05 was considered significant.

## Results

### The chemotherapy sensitization effect of TNF-α against breast cancer cells and xenografts

**Fig. 1:**
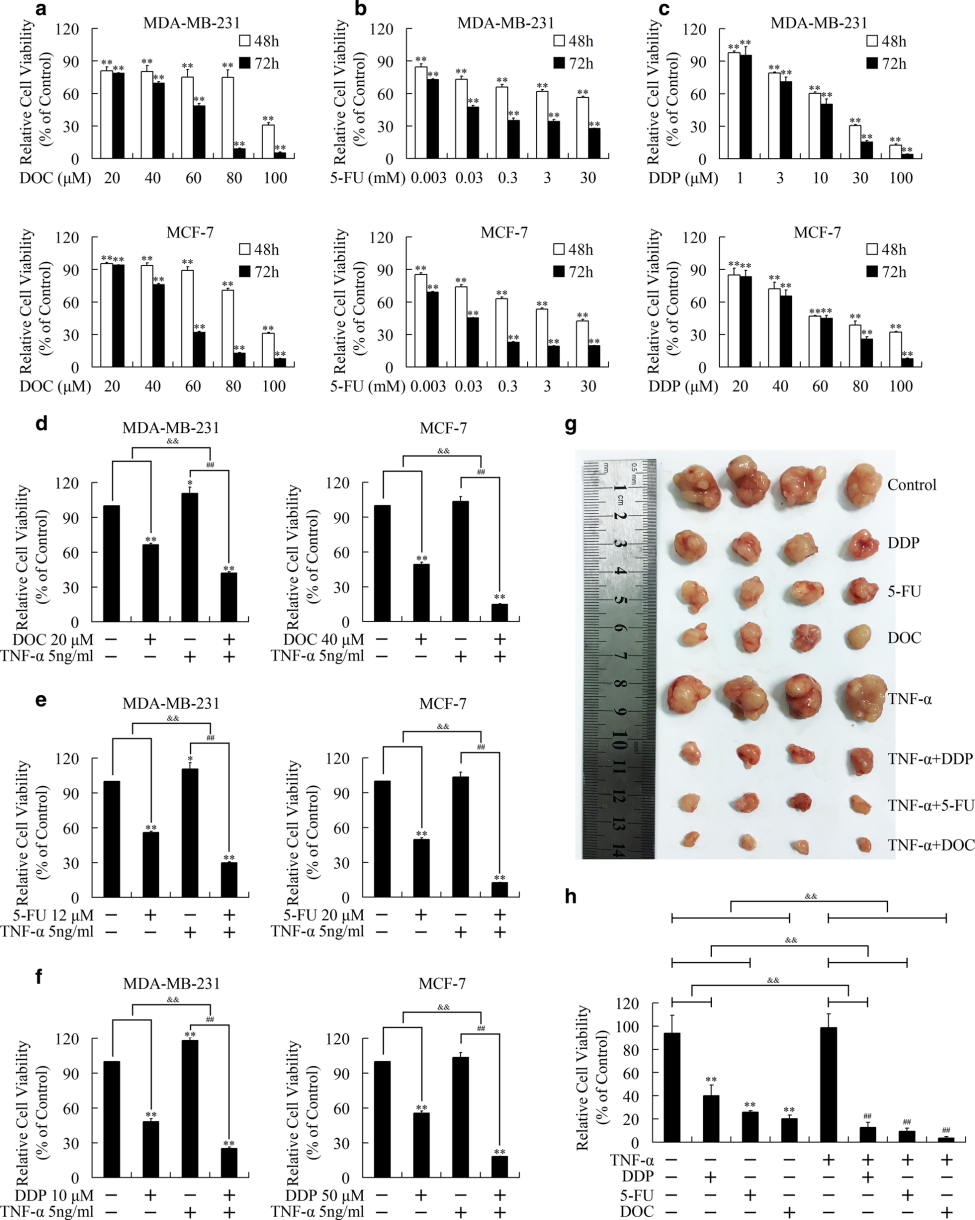
TNF-α sensitized cytotoxicity of docetaxel (DOC), 5-flurouracil (5-FU) and cisplatin (DDP) against MDA-MB-231 and MCF-7 breast cancer cells. **a**–**c** Dose- and time-dependent cytotoxicity of docetaxel (DOC) (**a**), 5-flurouracil (5-FU) (**b**) and cisplatin (DDP) (**c**). **P < 0.01 indicated significant differences from the respective control groups. **d**–**f** Enhanced cytotoxicity of docetaxel (DOC) (**d**), 5-flurouracil (5-FU) (**e**) and cisplatin (DDP) (**f**) with combined treatment of TNF-α (5 ng/ml). *P < 0.05 and **P < 0.01 indicated significant differences from the respective control groups; ^#^ *P* < 0.05 and ^##^ *P* < 0.01 indicated significant differences from the TNF-α (5 ng/ml) treated groups; ^&&^ *P* < 0.01 indicated significant differences between folds induction. **g**, **h** TNF-α (20 ng each injection, intratumorally, every 3 days) strengthened growth inhibition effect of docetaxel (DOC, 2 mg/kg, intravenously, every 3 days), 5-flurouracil (5-FU, 10 mg/kg, intraperitoneally, everyday) and cisplatin (DDP, 2 mg/kg, intraperitoneally, every 3 days) against breast cancer xenografts in vivo. **g** Image of stripped xenografts.** h** Tumor weight of each group. **P < 0.01 indicated significant differences from control group; ^##^ *P* < 0.01 indicated significant differences from TNF-α treated group; ^&&^ *P* < 0.01 indicated significant differences between folds induction

The growth inhibition effects of chemotherapeutics, docetaxel, 5-FU and cisplatin, against breast cancer cells were firstly evaluated by using MTT assays. As shown in Fig. 1a–c, docetaxel, 5-FU and cisplatin repressed growth of MDA-MB-231 and MCF-7 cells through dose- and time-dependent manner. The IC_50_ values, 48 and 72 h post treatment with each chemotherapeutic, were presented in Table 1.

**Table 1:** The IC_50_ values of docetaxel (DOC), 5-Fluorouracil (5-FU) or cisplatin (DDP) in MDA-MB-231 and MCF7 cells

	MDA-MB-231		MCF7	
	48 h	72 h	48 h	72 h
DOC (μM)	75.35	45.45	83.64	47.74
5-FU	119.33 mM	89.22 μM	5.75 mM	31.98 μM
DDP (μM)	13.48	8.61	59.71	47.39

According to IC_50_ values of chemotherapeutics, chemotherapy sensitization effect of TNF-α was evaluated in vitro. Co-treatment with TNF-α strengthened the growth inhibition induced by docetaxel, 5-FU and cisplatin remarkably (Fig. 1d–f). The chemotherapy sensitization effect was then assessed in vivo. As shown in Fig. 1g, h, TNF-α strengthened the suppression on the formation of xenografts by chemotherapeutics, confirming that TNF-α preserved synergetic effect on breast cancer growth with chemotherapeutics.

### The radiotherapy sensitization effect of TNF-α against breast cancer cells

**Fig. 2:**
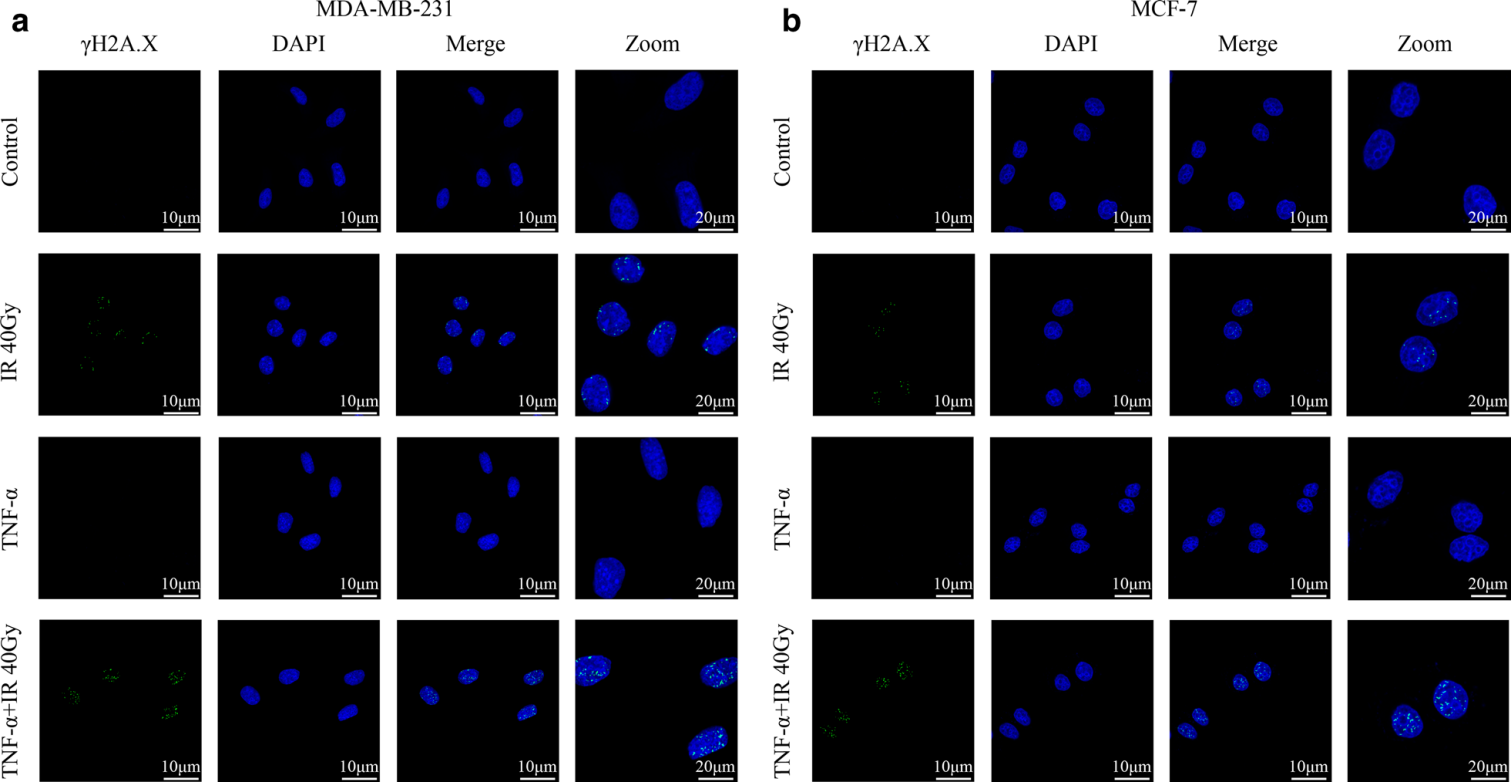
The radiotherapy sensitization effect of TNF-α. Formation of phosphorylated H2A.X (γ-H2A.X), a marker for DNA damage, was visualized in MDA-MB-231 (**a**) and MCF-7 (**b**) cells 30 min after a dose of 40 Gy Cs^137^-irradiation

To verify the radiotherapy sensitization effect of TNF-α, we measured the dynamic formation of phosphorylated H2A.X (γ-H2A.X), a marker of DNA double-strand breaks (DSBs) [20–22], in breast cancer cells after a dose of 40 Gy Cs^137^-irradiation. As shown in Fig. 2, the number of γ-H2A.X foci increased 30 min post irradiation. TNF-α treatment up-regulated γ-H2A.X foci formation to higher levels, suggesting the co-treatment of TNF-α brought more DNA damages.

### TNF-α upregulated cyclin D1, Cyclin D2, Cyclin E, CDK4 and CDK6 through activating NF-κB pathway, and accelerated G1→S cell cycle transition

**Fig. 3:**
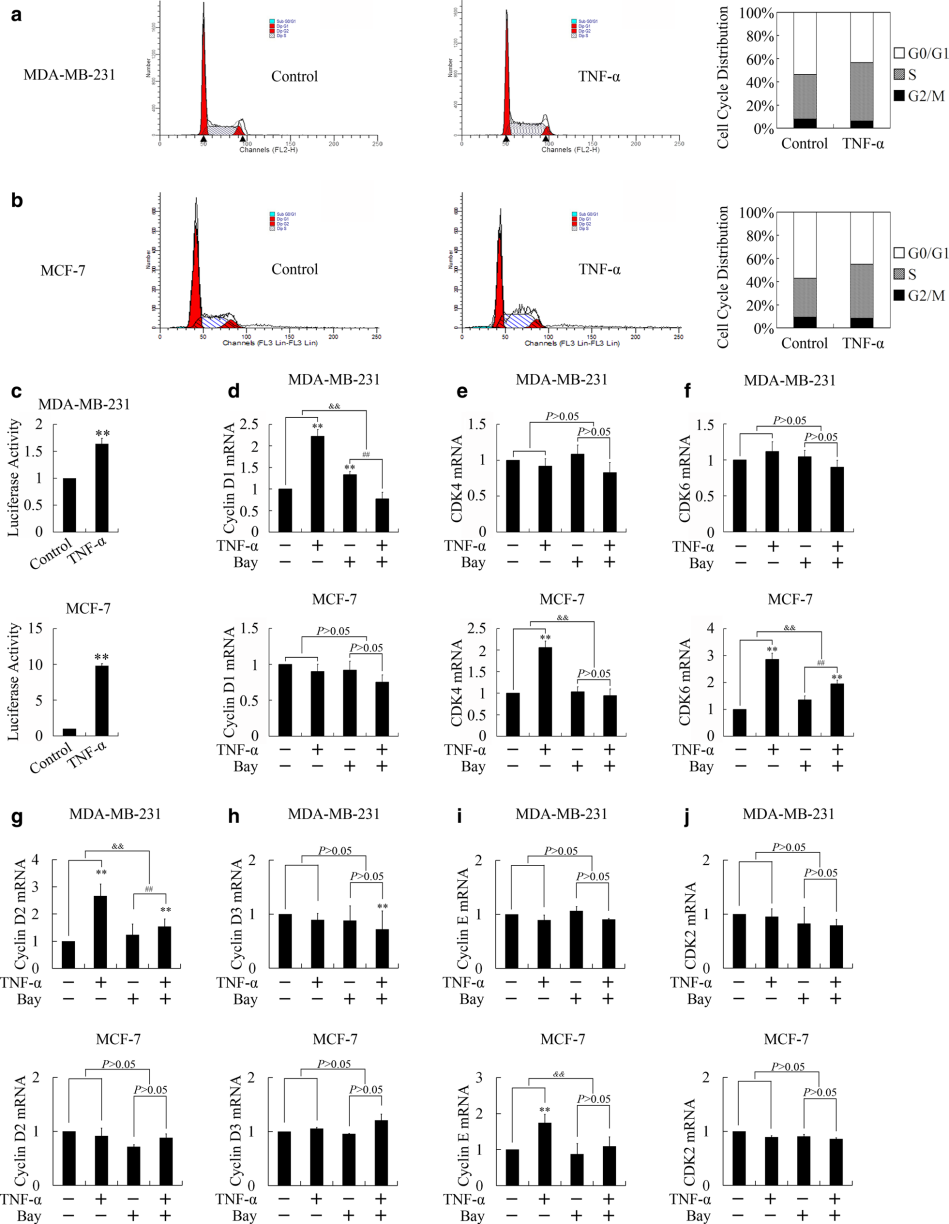
TNF-α promoted G1→S cell cycle transition through NF-κB pathway dependent manner. **a**, **b** Effects of TNF-α (5 ng/ml) on cell cycle distribution in MDA-MB-231 (**a**) and MCF-7 (**b**) cells 24 h post treatment. **c** Relative luciferase activity of NF-κB after treatment with TNF-α (5 ng/ml) for 24 h. **P < 0.01 indicates significant differences from the respective control groups. **d**–**j** Expressions of Cyclin D1 (**d**), CDK4 (**e**), CDK6 (**f**), Cyclin D2 (**g**), Cyclin D3 (**h**), Cyclin E (**i**), and CDK2 (**j**) after treatment of TNF-α (5 ng/ml) and/or NF-κB inhibitor, Bay11-7082 (Bay, 20 μM). **P < 0.01 indicates significant differences from the respective control groups; ^##^ *P* < 0.01 indicated significant differences from the TNF-α (5 ng/ml) treated groups; ^&&^ *P* < 0.01 indicated significant differences between folds induction

Cancer cells in G0/G1 cell cycle have been proved to be quiescent and resistant to chemotherapy and radiotherapy. Since TNF-α sensitized chemotherapy and radiotherapy, we then performed cell cycle analysis to explore the effect of TNF-α on cell cycle distribution. As shown in Fig. 3a and b, treatment with TNF-α induced remarkable decreased G0/G1 phase population and increased S plus G2/M phase proportion. These results indicated that TNF-α could drive cells out of quiescent G0/G1 phase, entering chemotherapy- and radiotherapy-sensitive proliferating phases.

Cyclin D1, Cyclin D2, Cyclin D3, Cyclin E, CDK2, CDK4 and CDK6 are the key regulators participating in G1→S phase transition, and NF-κB is the main pathway at the downstream of TNF-α stimulation. Therefore, we then investigate whether TNF-α could regulate expressions of G1→S phase transition regulators through NF-κB pathway dependent manner. Activation of NF-κB pathway was firstly confirmed by luciferase report gene assays (Fig. 3c). Treatment of TNF-α up-regulated Cyclin D1 and Cyclin D2 in MDA-MB-231 cells, and increased expressions of Cyclin E, CDK4 and CDK6 in MCF-7 cells (Fig. 3d–j). Pre-treatment of Bay11-7082, an inhibitor of NF-κB pathway, attenuated the up-regulation of Cyclin D1, Cyclin D2, Cyclin E, CDK4 and CDK6 (Fig. 3d–j), suggesting TNF-α up-regulated key regulators participating in G1→S cell cycle transition through NF-κB pathway dependent manner.

### TNF-α strengthened cell cycle arrest induced by docetaxel and cisplatin

**Fig. 4:**
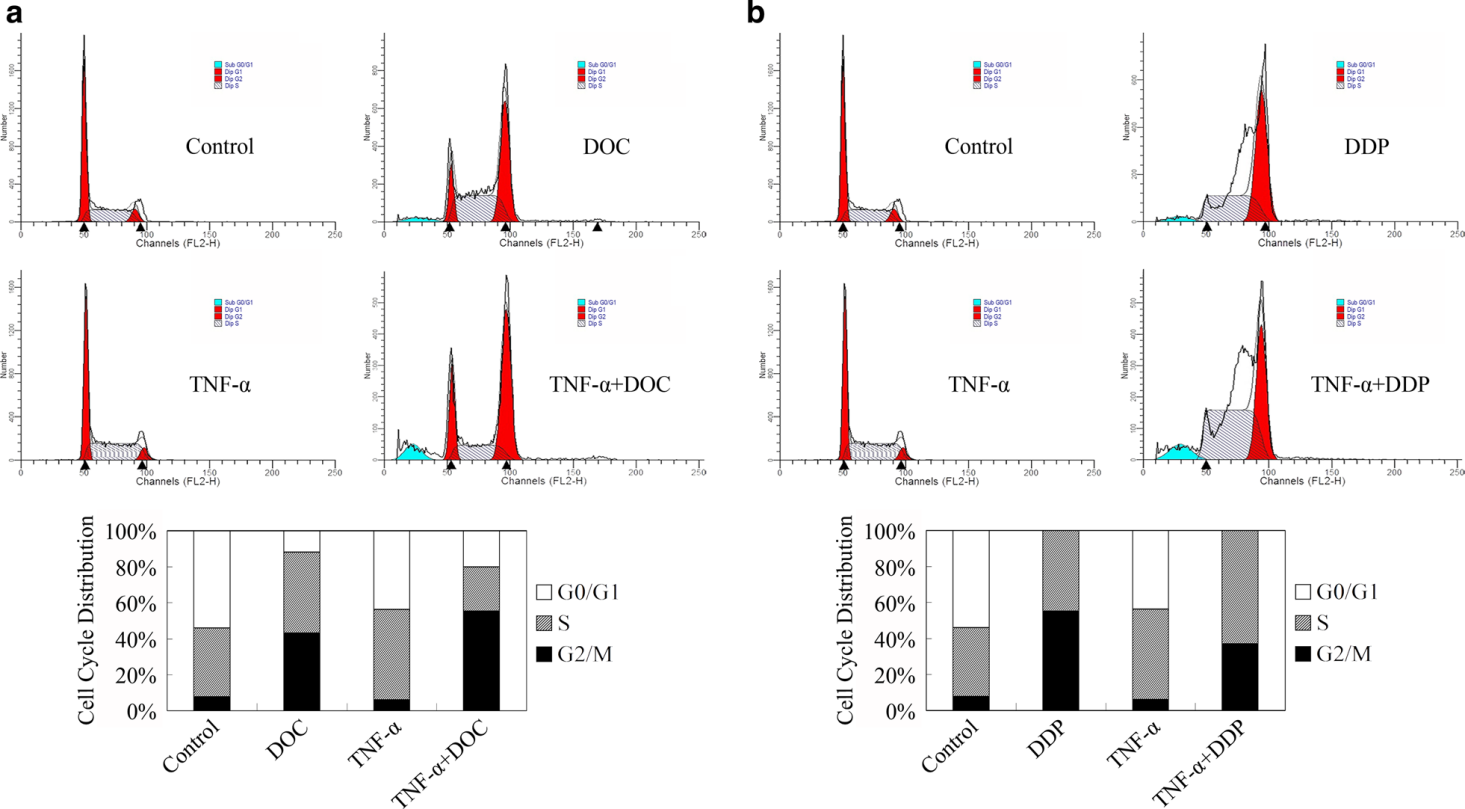
TNF-α strengthened cell cycle arrest induced by docetaxel (DOC) and cisplatin (DDP) in MDA-MB-231 breast cancer cells. **a** Cell cycle distribution after treatment with TNF-α (5 ng/ml) and/or docetaxel (20 μM). **b** Cell cycle distribution after treatment with TNF-α (5 ng/ml) and/or cisplatin (10 μM)

We then investigated whether the chemotherapy sensitization effect could involve the regulation on cell cycle distribution. As shown in Fig. 4a, docetaxel induced G2/M cell arrest and decreased population of cells at G0/G1 plus S phases, consisting with its microtubule structure stability role [23]. Co-treatment with TNF-α increased G2/M cell cycle arrest which was induced by docetaxel.

Cisplatin induced accumulation of cells at S phase in Fig. 4b, due to its DNA crosslinking role [24]. Co-treatment with TNF-α increased S phase cell cycle arrest induced by cisplatin.

Therefore, TNF-α drove cells out of G0/G1 phase, leading more cells entering S and G2/M phase and these proportions were further arrested by cisplatin and docetaxel at S and G2/M phases respectively.

### TNF-α increased necroptosis induced by 5-FU

**Fig. 5:**
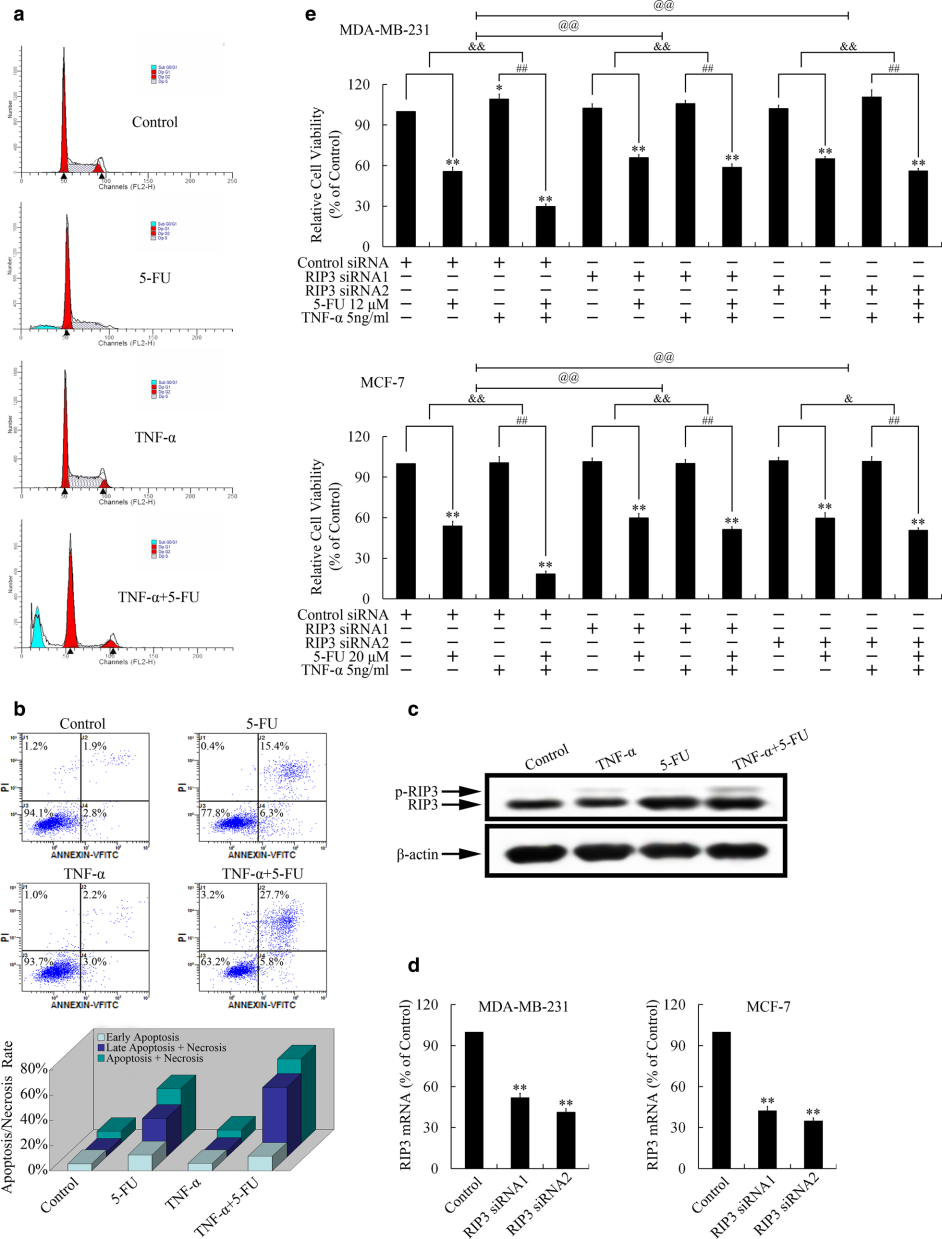
Co-treatment of TNF-α and 5-FU induced necroptosis in MDA-MB-231 breast cancer cells. **a** Cell cycle distribution after treatment with TNF-α (5 ng/ml) and/or 5-FU (12 μM). **b** Apoptosis/necrosis rate after treatment with TNF-α and/or 5-FU. **c** Protein level of necroptosis marker, RIP3, after treatment with TNF-α and/or 5-FU. **d** Confirmation of knockdown of RIP3 by using real-time PCR. **e** Effect of RIP3 knockdown on the synergetic effect of TNF-α and 5-FU. **P < 0.01 indicated significant differences from the respective control groups; ^##^ *P* < 0.01 indicated significant differences from the TNF-α (5 ng/ml) treated groups; ^&^ *P* < 0.05, ^&&^ *P* < 0.01 and ^@@^ *P* < 0.01 indicated significant differences between folds induction

Figure 5a revealed that 5-FU caused G0/G1 cell arrest through its inhibition of thymidylate synthase [25]. Therefore, TNF-α might not be able to drove cells out of G0/G1 phase in this circumstance. However, combined application of TNF-α with 5-FU induced a significant sub G0/G1 peak, suggesting necrosis and/or apoptosis induction might be involved in the TNF-α-induced 5-FU sensitization.

Therefore, we performed apoptosis assays to further investigate the mechanisms involved. The percentages of cell populations at various stages of apoptosis are shown in Fig. 5b. After co-treatment with TNF-α and 5-FU, the numbers of cells that underwent later apoptosis and/or necrosis (Annexin V+/PI+) increased significantly. These data suggested that the enhanced growth-inhibition effect of TNF-α combined with antitumor drugs could be due to induction of later apoptosis and/or necrosis.

It has been reported that necrosis induced by TNF-α is distinguished from traditional necrosis, and termed as necroptosis (programmed necrosis) [26]. Therefore, we evaluated the activation of necroptosis marker, receptor-interacting protein kinase 3 (RIP3), by using Western blot. As shown in Fig. 5c, TNF-α treatment induced phosphorylation, the active form of RIP3 [26]. 5-FU had no effect on RIP3 phosphorylation, but up-regulated expression of RIP3. Therefore, co-treatment of TNF-α and 5-FU led to significant activation of RIP3, suggesting the involvement of necroptosis in the 5-FU sensitization effect of TNF-α.

To verify whether the chemotherapy sensitization effect of TNF-α on 5-FU was executed through a RIP3-dependent manner, we applied siRNAs targeting RIP3 (Fig. 5d). As expected, knockdown of RIP3 attenuated the synergetic effect of TNF-α and 5-FU (Fig. 5e), indicating TNF-α strengthened cytotoxicity of 5-FU through RIP3-dependent necroptosis.

## Discussion

Resistance to anticancer drugs and radiotherapy is the primary reason for treatment failure in cancer [27]. Cytotoxic agents and radiotherapy killed only proliferating cancer cells and, in contrast, had little effect on quiescent cancer cells [28]. After cessation of chemotherapy and radiotherapy, these therapeutic-resistant quiescent cancer cells restarted cycling, leading to a relapse situation. Therefore, drugs that target quiescent cancer cells are urgently needed. In our present study, we proved that, TNF-α preserved the radiotherapy-sensitizing ability and strengthened cytotoxicity of chemotherapeutics, including docetaxel, cisplatin and 5-FU, making TNF-α a promising candidate for further clinical application.

Mitotic cellular division requires the cell to leave the resting state and proceed through phases of DNA synthesis and mitosis. Well-organized progression of dividing cells through the G0, G1, S, G2, and M phases of the cell cycle in eukaryotic cells relies on a series of cell-cycle regulatory proteins, such as Cyclin A, B, D and E, and these cyclins exert their functions via binding to and activating a variety of specific cyclin-dependent kinases (CDKs) [29]. Furthermore, there are a number of kinases and phosphatases which modulate CDK respectively through a way of phosphorylation and dephosphorylation. For instance, the regulation of the CDKs activity is associated with many CDK inhibitors (CKIs), and cell cycle checkpoint proteins. Cell division is conceived as a passage through these checkpoints. The earliest of these is the ‘‘restriction point’’ late in G1, when the cell becomes irreversibly transformed to DNA synthesis and mitosis. Cyclin D1 is a key regulator of G1/S checkpoint control, which forms a holoenzyme complex with CDK4 and CDK6, to phosphorylate pRb (retinoblastoma protein) [30]. When phosphorylated, pRB releases transcription facctor E2F to promote cell cycle [29, 31]. Previous studies have shown that the Cyclin D1 promoter region includes binding sites for NF-κB [32], and NF-κB is required to induce Cyclin D1 expression and pRb hyperphosphorylation to promote G1→S transition. There are other two D group of cyclins, Cyclin D2 and Cyclin D3. Cyclin D2 abundance peaks in late G1-phase. And cyclin D3 peaks during S-phase. Like cyclin D1, both Cyclin D2 and Cyclin D3 associate with CDK4 and CDK6, forming holoenzyme complexes that phosphorylate pRB [33]. Cyclin E, which forms a complex with CDK2, reaches abundance peak in late G1-phase [34]. The Cyclin E/CDK2 complex also phosphorylates pRB, although some phosphorylation sites are distinct from those targeted by kinases associating with D group cyclins. Consistent with our results in Fig. 3, apparently, the activation of NF-κB by the pre-treatment of TNF-α stimulates transcriptions of Cyclin D1, Cyclin D2, Cyclin E, CDK4 and CDK6 to accelerate the progression of cell cycle from G0/G1 phase to S phase. As a result, TNF-α drove quiescent cancer cells out of G0/G1 phase, entering treatment sensitive proliferating phases. By using cell cycle analysis, we further confirmed that TNF-α strengthened the G2/M phase and S phase cell cycle arresting ability of docetaxel and cisplatin respectively.

Actually, TNF-α is a pleiotropic cytokine that plays a vital role in a wide variety of physiological processes [35]. The pleiotropic nature of TNF-α response results from the sequential formation of different signaling complexes via binding of TNF-α to its receptor. So far, there are two identified TNF-α receptors, TNF-α receptor 1 (TNFR1) and TNF-α receptor 2 (TNFR2), both of which belong to the TNFR superfamily. TNFR1 has a single intracellular death domain (DD), whereas TNFR2 has a TNF receptor-associated factor (TRAF)-binding area [36]. There is a broad body of evidences that TNF-induced cell death is largely mediated via TNFR1 [6, 8, 10, 23]. Binding to TNFR1 can cause apoptosis by caspase 8 or necroptosis by receptor-interacting protein kinase 1 (RIP1)- and RIP3-dependent mechanisms [36–38]. Necroptosis, or programmed necrosis, refers to a caspase-8-independent death mechanism triggered by RIP1 and RIP3. RIP1 bears a DD that can interact with TNFR1, upon binding of TNF-α, RIP1 is recruited to TNFR1 either directly via its DD or indirectly by TRADD, to form a complex on the cytoplasmic domain of TNFR1 [39]. The complex is released from TNFR1 and RIP3 to cause necroptosis. If cells have high level of RIP3, RIP1 recruits RIP3 to form necrosome containing FADD [40, 41], caspase-8, RIP1, and RIP3, and the cells undergo necroptosis. Necrosome also suppresses apoptosis but the underlying mechanism has not been described yet. Mixed-lineage kinase domain-like (MLKL) is downstream of RIP3 [42, 43], the phosphorylated RIP3 recruits MLKL, and phosphorylation of MLKL is required for subsequent execution of necroptosis. Therefore, RIP3 is conceived as a key regulator of necroptosis.

5-FU can suppress thymidylate synthase and block DNA synthesis, causing a restraint of the transition from G1 phase to S phase [25]. Although the G0/G1 cell cycle arrest effect of 5-FU may antagonise the G1→S transition accelerating effect of TNF-α, co-treatment with TNF-α still strengthened the cytotoxicity of 5-FU, suggesting mechanisms beyond cell cycle regulation might be involved. It was noteworthy that there was a significant peak in sub G0/G1 phase in the co-treating group. Therefore, we carried out the apoptosis assays and detected a remarkble Annexin V+/PI+ population in the combination group of TNF-α and 5-FU, suggesting an apoptosis and/or necroptosis mechanism might be involved. We further demonstrated that 5-FU was able to up-regulate RIP3 expression. Since TNF-α activates RIP3 and induce necroptosis, the synergistic effect of co-treatment of TNF-α and 5-FU might be executed through elevated necroptosis. In consisting with this assumption, RIP3 siRNA apparently attenuated the synergistic effect by the combination of TNF-α and 5-FU.

In conclusion, we presently proved that, TNF-α present radiotherapy- and chemotherapy-sensitizing effect against breast cancer cells. TNF-α drove cells out of quiescent G0/G1 phase, entering vulnerable proliferating phases. As a result, TNF-α brought more DNA damage after irradiation and strengthened G2/M and S phase cell cycle arrest induced by docetaxel and cisplatin respectively. Moreover, the up-regulation of RIP3 by 5-FU, and the activation of RIP3 by TNF-α, synergistically triggered necroptosis. Taking together, our present investigation brought new strategies for breast cancer treatment through sensitized chemotherapy and radiotherapy by TNF-α.

## Conclusions

TNF-α presented radiotherapy- and chemotherapy-sensitizing effects against breast cancer cells.

## Abbreviations

PCD: programmed cell death
DOC: docetaxel
5-FU: 5-flurouracil
DDP: cisplatin
MTT: methyl thiazolyl tetrazolium
DMSO: dimethyl sulfoxide
PI: propidium iodide
LTα: lymphotoxin-α
FasL: fas ligand
CD40L: CD40 ligand
TRAIL: TNF-related apoptosis-inducing ligand
TNFR1: TNF-α receptor 1
TRAF: TNF receptor-associated factor
TRADD: TNF receptor-associated death domain
RIP1: receptor-interacting protein 1
NF-κB: nuclear factor of kappa light polypeptide gene enhancer in B-cells
CDKs: cyclin-dependent kinases
CKI: cyclin-dependent kinase inhibitor
CDC: cell division cycle protein
DD: death domain
FADD: Fas-associated with death domain protein
DISC: death-inducing signaling complex
pRb: retinoblastoma protein
RIP: receptor-interacting protein kinase
MLKL: mixed-lineage kinase domain-like
